# Footprints of Urban Micro-Pollution in Protected Areas: Investigating the Longitudinal Distribution of Perfluoroalkyl Acids in Wildlife Preserves

**DOI:** 10.1371/journal.pone.0148654

**Published:** 2016-02-24

**Authors:** Ignacio A. Rodriguez-Jorquera, Cecilia Silva-Sanchez, Mark Strynar, Nancy D. Denslow, Gurpal S. Toor

**Affiliations:** 1 Interdisciplinary Ecology Program, School of Natural Resources and Environment, Soil and Water Science Department, University of Florida, Gainesville, Florida, United States of America; 2 Department of Physiological Sciences & Center for Environmental and Human Toxicology, University of Florida, Gainesville, Florida, United States of America; 3 Proteomics and Mass Spectrometry, Interdisciplinary Center for Biotechnology Research, University of Florida, Gainesville, Florida, United States of America; 4 United States Environmental Protection Agency, National Exposure Research Laboratory, Durham, North Carolina, United States of America; 5 Soil & Water Quality Laboratory, Gulf Coast Research & Education Center, University of Florida, Institute of Food & Agricultural Sciences, Wimauma, Florida, United States of America; University of Wisconsin Milwaukee, UNITED STATES

## Abstract

Current approaches to protect biodiversity by establishing protected areas usually gloss over water pollution as a threat. Our objective was to determine the longitudinal and seasonal distribution of perfluoroalkyl acids (PFAAs) in water column and sediments from a wastewater dominated stream that enters preservation areas. Water samples were collected along the longitudinal section (six sites, 1000 m away from each other) of the stream during the dry and wet seasons. Sediments were collected from three sites along the stream from three depths. Water and sediments were analyzed for PFAAs using high performance liquid chromatography-tandem mass spectrometry. Eleven PFAAs with 5 to 14 carbon atoms were detected in the water column at all sampling points, with a minor reduction at the last point suggesting a dilution effect. The most detected PFAAs was PFOS, followed by perfluorooctanoic acid (PFOA), and perfluorohexanoic acid (PFHxA). Seasonal differences in PFAAs concentrations suggested contribution of stormwater runoff during the wet season. All analyzed PFAAs in sediments were under the limit of quantification, likely due to the high proportion of sand and low organic matter. However, high concentrations of PFAAs were detected in the water column inside the protected areas, which includes PFOS in concentrations considered not safe for avian wildlife. Water samples appear to be more relevant than sediments to determine PFAAs micro-pollution in water bodies with sandy sediments. Inclusion of a management plans on micro-pollution research, monitoring, and mitigation is recommended for protected areas.

## Introduction

Ecological degradation is consistently observed in urban streams. Stressors such as a combination of sanitary sewer overflows and effluents from wastewater treatment plants (WWTPs) are listed as main causes of urban streams impairment [1]. Historically and still today, nutrients (nitrogen and phosphorus) are the major pollutants discharged in wastewater from WWTPs [2]. As such, WWTPs are now required to meet nutrient threshold limits when discharging wastewater into surface water bodies. The use of WWTPs to control the point sources of pollution positively changed the quality of surface waters as well as helped restore aquatic biodiversity [3]. However, it is now known that many organic contaminants including perfluoroalkyl acids (PFAAs) are not effectively removed in WWTP and persist in discharged wastewater [4]. Pollution of long chain PFAAs is a globally prevalent environmental issue of concern [5].

Various sources of the highly persistent, bioaccumulative and toxic PFAAs in the aquatic systems include WWTPs, leachate from landfill, and stormwater runoff from streets [6–8]. WWTPs are considered a major source of PFAAs, particularly those that treat industrial effluents [9, 10]. It is well known that PFAAs are chemicals with long environmental persistence and are not removed in WWTPs [11–13]. Further, WWTPs can augment (in-plant production) the amount of PFAAs in wastewater due to degradation of their precursor compounds [14]. However, the net mass balance increase of PFAAs in WWTPs effluent depends on PFAAs functional groups [15]. For example, in the WWTPs effluent, the concentration of perfluorooctanane sulfonic acid (PFOS) tends to decrease while perfluoro octanoic acid (PFOA) increases [8, 15, 16]. In general, PFAAs input from WWTPs to the receiving water bodies seems to remain constant throughout the year, as no major seasonal changes in their concentrations have been observed in research conducted in wastewater dominated aquatic systems [17], although some seasonal variations have been observed particularly for PFOS [18].

In sediments, the sorption behavior of PFAAs has been investigated in terms of the solid to water partition coefficient (K_d_) [19–21], where chain length and functional group of PFAAs have a pronounced effect on their partitioning. Thus, PFAAs with carbon (C) chain lengths (C<7) are exclusively found in the dissolved phase, while long chain (C>7) appeared to bind to particles [19]. Short-chain PFAAs have a higher potential for transport in water, while the long-chain tend to be distributed in biota or the abiotic environment like sediment, which could act as a sink for PFAAs [10, 22]. Previous work demonstrated that chain length was the dominant structural feature influencing PFAAs sorption onto river sediments [19]. Moreover, organic C is a dominant parameter affecting sorption of PFAAs in sediments [19].

Due to the highest global occurrence and persistence of dominant types of PFAAs (i.e. PFOA and PFOS) in various environmental matrices including water and sediments [5], monitoring the presence of an expanded group of PFAAs is necessary to understand their impacts on ecological health [23]. Further, in the context of urban expansion and the corresponding impact on protected areas [24], it is important to protect the areas set aside as nature preserves. Traditionally, within the known threats that preserved areas face, pollution is mentioned [25] but in depth assessment is typically missing. Our planet has 209,429 protected areas covering 32,868,673 km^2^ area which protects 14% of the world’s terrestrial surface [26]. Nationally, the USA has 22,599 protected areas covering 12% of terrestrial land area in the country [27].

**Fig 1:**
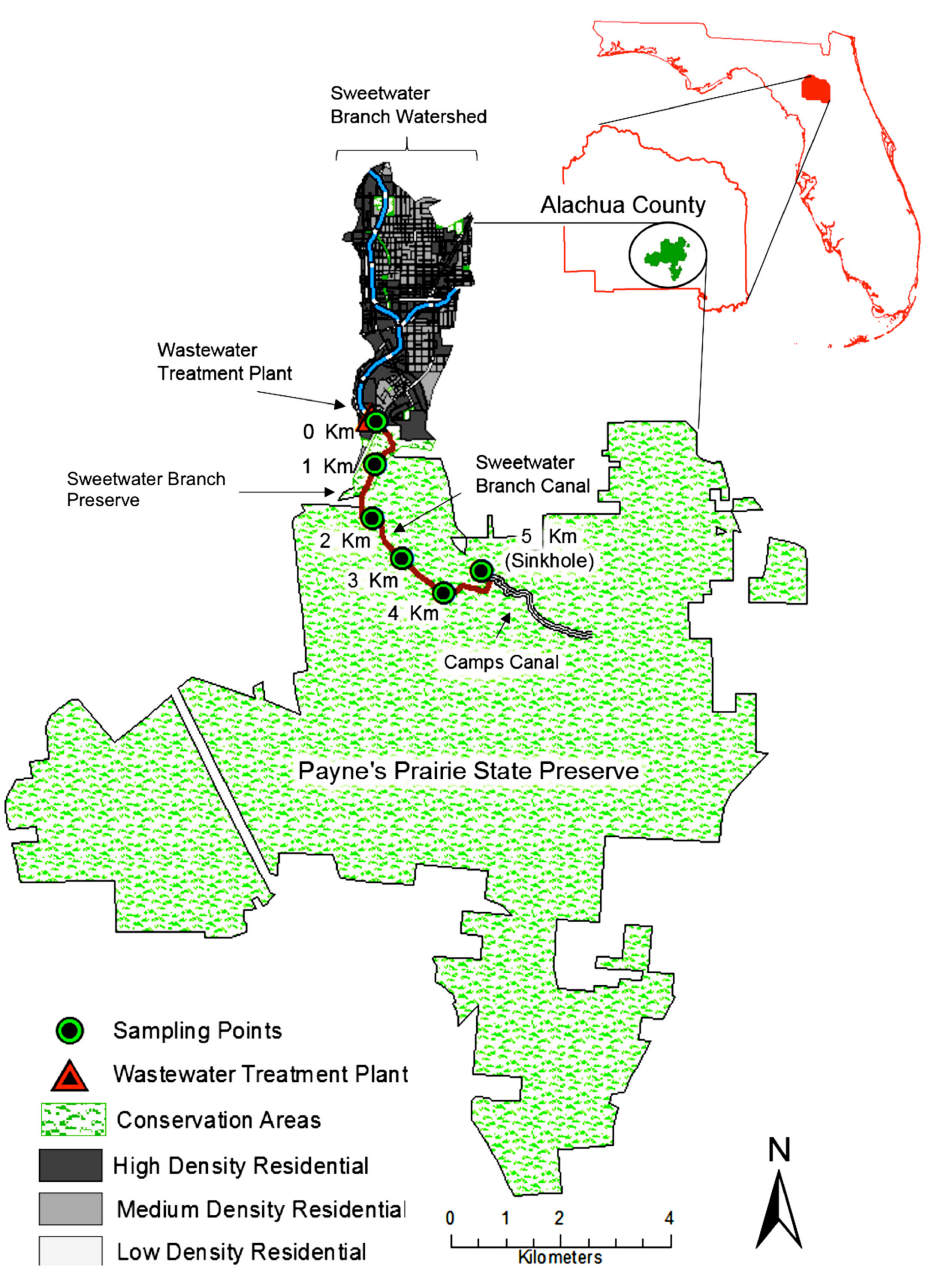
Water and sediment sampling points along Sweetwater branch canal.

In Florida, the location of this study, the system of parks and recreation areas aims to provide resource-based recreation, while preserving and restoring natural resources. Six State parks are located in the Alachua County boundary (Florida) including two preserve parks. Treated wastewater from a WWTP is discharged into Sweetwater branch, a highly modified stream that enters first into the relatively small (50 ha) Sweetwater Preserve, then further flows into the county’s larger (8,498 ha) preservation area: Payne’s Prairie State Park (Fig 1). Our previous work showed the presence of PFAAs and several organic contaminants in the Sweetwater branch [28]. The finding of relatively high concentrations of the persistent PFAAs and their potential toxicological concerns prompted the need to investigate the longitudinal occurrence of PFAAs from the source (WWTP) to sink (where surface water flows to groundwater) to determine their potential impacts on aquatic and terrestrial biota. Our hypothesis was that due to the high persistence of PFAAs in the environment, their concentrations would be similar longitudinally in water and sediments from source to final destination in a stream that passes throughout two protected areas. Our overall aim was to demonstrate that fingerprints of urban micro-pollution are present in protected areas in concentrations considered harmful to certain aquatic and terrestrial organisms.

## Materials and Methods

### Study Site

Samples were taken from the highly modified Sweetwater branch stream located in the Payne’s Prairie sub-basin in Gainesville, Alachua County, Florida, United States. The Sweetwater Branch enters into two wildlife preservation areas: Sweetwater Preserve and Payne’s Prairie Preserve State Park. Average annual precipitation for Gainesville city area is 121 cm (NOAA, 1981 to 2010 [29]). More than half of this amount falls from June to September. August, the month with the greatest precipitation, averages 16 cm, while the least precipitation occurs in November. Yearly temperatures range from an average low of 14.2°C to an average high of 26.6 (°C). Payne's Prairie is a 5,600 ha sub-basin resulting from dissolution of the underlying limestone [30], located 16 km south of Gainesville city. The presence of limestone beneath much of the surface has resulted in the formation of sinkholes, large shallow lakes and broad wet prairies [30]. Sources of water flowing into the basin include the Sweetwater branch (Fig 1) that drains eastern part of Gainesville and has along its course a WWTP; all the flow then enters directly into La Chua Sink [30]. As sampling in the preserved areas is restricted, we obtained all required permits from the protected areas officials and from the Florida Fish and Wildlife Conservation Commission prior to the beginning of the study.

### Water Samples Collection

Grab surface water samples were collected two times each in the dry (October-May) and wet (June-September) seasons during 2012–2013. To cover the longitudinal area along the Sweetwater branch, a sampling point was set at every 1 km (Table 1), starting immediately downstream of a WWTP (point 0) and finishing at the La Chua Sink (point 5), as shown in Fig 1. This resulted in six sampling points along the 5 km stretch of the stream. A total of 48 samples were collected (6 sites x 2 samples per site x 4 times in a year). At each sampling site, 1 L water was collected, in duplicate, in pre-washed polypropylene bottles because PFAAs may be sorbed onto glass surfaces [31]. Before sampling, all bottles were washed three times with soap, then three times with deionized water, and finally with HPLC grade methanol. A preservation agent was prepared and added prior to sample collection, as indicated in Shoemaker et al. (2009) [32]: This included a customized blend of Tris [Tris (hydroxymethyl) amino methane] and Tris HCL [Tris (hydroxymethyl) amino methane hydrochloride] with a weight ratio of 15.5/1. Five grams of this blend was added into cleaned bottles before sampling to produce a pH near 7.0 at 25°C. This blend functions as a buffer and removes the free chlorine in chlorinated waters such as treated wastewater [32, 33].

**Table 1:** Geographic coordinates for each of the sampling points in this study.

Site	N	W
0K	29°37'49''	82°19'20''
1K	29°37'23''	82°19'20''
2K	29°37'54''	82°19'27''
2K^DP^	29°37'49''	82°19'18''
3K	29°36'33''	82°19'07''
4K	29°36'12''	82°18'39''
5K (Sink)	29°36'23''	82°18'11''

Samples were then collected 10 cm below the surface of flowing water from all the six sites. A field blank made with Milli Q^**®**^ water was taken at the last sampling site during each sampling time and was processed similar to other samples. All samples were transported on ice to the laboratory, stored at -20 C°, and analyzed within 2-weeks of the sampling date. No filtration step was required for water samples as there was no visible suspended particulate matter, thus solid-phase extraction (SPE) cartridges were not clogged. Historical data that includes other organic pollutants besides PFAAs was obtained from Alachua Environmental Protection Department [28, 34].

### Extraction of PFAAs in Water Samples

Eleven PFAAs were analyzed using high performance liquid chromatography coupled with tandem mass spectrometry (HPLC/ESI-MS/MS) [33]. In brief, 11 native PFAAs and mass-labeled PFAAs were purchased from Wellington Laboratories Inc. (Guelph, Ontario, Canada). Mass labeled perfluoroalkyl carboxylic acid (MPFOA: perfluoro-n-[1,2,3,4-^13^C4] octanoic acid) and perfluoroalkyl sulfonate (MPFOS: sodium perfluoro-1-[1,2,3,4-^13^C4] octanesulfonate) were used as internal standards (IS). A 40 ng/L of MPFOA was added to each sample immediately before SPE. The Oasis^®^ HLB 6 cm^3^ cartridges (Waters Corp. Milford, MA, USA) with 200 mg sorbent per cartridge (30 μm particle size) were used for the SPE of the water samples.

The conditioning of the cartridge was performed with 15 mL of methanol followed by 18 mL of reagent water, without allowing the water to drop below the top edge of the packing. Polypropylene tubing (also pre-washed with methanol) was used to transfer the water from bottles to cartridges. The vacuum was adjusted in the manifold to allow approximately two drops of water per second. After the entire sample was passed, two 5 ml of reagent water was used to rinse sample bottles and passed through the transfer tubes. The elution of cartridge was performed using 4 ml of methanol added to the bottles and pulled through the transfer tubes; this was repeated two times for each sample. The extract was concentrated to dryness with nitrogen gas in a heated water bath (60°C). The recovery percentage of PFAAs from water samples ranged from 67 to 127% (average: 101.8%, SD = 9.1), which is within the accepted extraction efficiency (between 50 to 150% of recovery) when using labeled PFAAs internal standards [32, 33].

### Analysis of PFAAs in Water Samples

Isotope dilution was used to determine PFAAs in water samples. After extraction and drying of water samples, samples were then solubilized in 1 mL of 96:4% (v/v) methanol: water containing 40 ng/mL of MPFOS and injected into the instrument for analysis. The analysis of PFAAs in water samples was performed using an Agilent 1100 series liquid chromatograph (Agilent Technologies, Wilmington, DE) coupled to a 4000 QTRAP mass spectrometer (AB SCIEX, Foster City, CA, USA). A C18 Vydac column (2.1 mm i.d. X 150 mm length, 5 μm) from Grace Davison Discovery Science (Deerfield, IL) was used. A Luna C18 column (4.6 mm i.d X 30 mm length, 3 um) was inserted between the pump and the injector to eliminate any background flourochemicals from poly (tetrafluoroethylene) instrument components.

A standard curve was prepared from 0 to 80 ng/mL of PFAAs; PFOS and PFOA remained constant at 40 ng/mL at all points of the curve. A 5 μL of each standard and sample was injected onto the column and PFAAs were separated using 2 mM ammonium acetate solution as solvent A, and methanol as solvent B at a flow rate of 300 nL/min. The initial condition was set at 40% of solvent B and held for 2 min, followed by a gradient from 40% to 80% of solvent B in 16 min, and held for 2 min. Solvent B was increased to 100% in 1 min and held for another 2 min to ensure that all compounds would elute. Finally solvent B was dropped to 40% in 1 min and held for 7 min to allow the reconditioning of the column for the next injection. Two replicates were injected per sampling point and after each sample at least two blanks were run to eliminate carry over between samples. The column elution was directly sprayed into the mass spectrometer using a turbo V ion spray interface operated in negative mode. The instrument parameters were set as follows: desolvation temperature 300°C, desolvation (curtain) gas 20 arbitrary units (AU), ion spray voltage -4500 V, nebulizer gas 1 and 2 were set at 20 AU respectively, CAD (Collisional activated dissociation) was set high, and dwell time was 40 msec. Mass spectrometry data were acquired in multiple reaction monitoring (MRM) mode. All instrument parameters for PFAAs standards were optimized and at least two MRM transitions per compound were used whenever was possible (S1 Table).

Quantitation of samples was performed in Analyst software (v1.5). The standard curve and samples were normalized using MPFOS as internal standard. Concentrations of samples were obtained by interpolating the normalized area ratios against the standard curve. The recovery rates were calculated by measuring the amount of added internal standard (MPFOS) after extraction.

### Sediments Collection and Analyses

A stainless steel corer was used to obtain the sediment samples from the center of the stream at three sites (points 0, 3, 5, as shown in Fig 1). Each core was divided into three depths of 0–5, 5–10, and 10–15 cm. The collected samples were placed in polypropylene jars to avoid cross contamination with PFAAs contained in other types of plastics and stored at 4°C.

Sediments were analyzed for pH (EPA 150.1), electrical conductivity (EPA 120.1), texture (sand, silt, clay), chloride (EPA 325.2), nitrogen forms (NH_4_-N EPA 350.1; NO_3_-N EPA 353.2; Total Kjeldahl N EPA 351.2) and metals (Mehlich 3 extraction, followed by EPA 200.7 for determination).

Sediment samples were extracted and analyzed for 10 PFAAs following the methods developed for soil analysis [35]. In brief, sediment samples were air dried (24 h) to remove excess water, and sampled (1 g) for extraction and analysis. Samples were subjected to methanolic sonic extraction (10 mL for 30 min), centrifuged (16,800 g) and the supernatant was passed through a pre-cleaned Supelclean ENVI-Carb SPE cartridge (Supelco, Bellefonte, PA). The methanol contained a suite of perfluorinated internal standards (^13^C_4_-PFBA, ^13^C_2_-PFHxA, ^18^O_2_-PFHxS, ^13^C_2_-PFOA, ^13^C_5_-PFNA, ^13^C_4_-PFOS, ^13^C_2_-PFDA) (Wellington Laboratories, Guelph, ON, Canada) for isotope dilution quantitation. The supernatant passing the cartridge was evaporated to ~1.0 mL (45°C) and prepared in 75:25 methanol: 2mM ammonium acetate in DI water. Standard curves were prepared from extracted standards (no blank matrix) and ranged from 0.1 to 50 ng/g sediment. Sediment extracts were analyzed using a Waters Acquity™ ultra performance liquid chromatograph interfaced with a Waters Quattro Premier XE triple quadrupole mass spectrometer (UPLC-MS/MS) (Waters, Milford, MA, USA). Samples were injected onto an Acquity UPLC® BEH C18 column (2.1 mm i.d. × 50 mm, 1.7 μm; Waters, Milford, MA, USA). Analytes were gradient separated with a mobile phase made up of 2 mM ammonium acetate aqueous solution with 5% methanol (solvent A) and 2 mM ammonium acetate in methanol (solvent B) over a 12 min gradient. A PFAAs isolator column (part of the Waters Acquity PFAAs Analysis Kit, Waters corporation Milford, MA, USA) was installed between the mixing chamber and injector to eliminate contamination resulting from buildup of PFAAs from the mobile phase on the head of the LC column during column equilibration. In doing so, systemic PFAAs contamination is chromatographically separated from analytical PFAAs. Electrospray negative ionization was used in the mass spectrometer source. The capillary voltage was set at negative 0.4 kV. Cone gas and desolvation gas flows were 0 and 1200 L/h, respectively. The source temperature was 150°C and the desolvation temperature was 350°C. Transitions for all ions were observed using MRM and analyte-specific mass spectrometer parameters were optimized for each compound. The recovery of PFAAs in the sediments ranged from 59 to 131% (average: 104.8, SD = 15.6), which was within the accepted extraction efficiency for sediments [33].

### Statistical Analyses

Analysis of variance (ANOVA) was used to determine if the concentration of each PFAAs was different longitudinally within a season and for each sampling point between the seasons (dry vs. wet). Fisher's least significant difference (LSD) method for multiple comparisons was used as a post hoc analysis to determine the specific longitudinal differences among sites within a season (wet or dry) and between seasons (wet vs. dry). The package “Agricolae” [36] from the open-source software “R” [37] was used to perform all calculations.

## Results and Discussion

The PFAAs can be directly or indirectly (from degradation products) released into the aquatic environment from manufacturing and/or from products that contain PFAAs [38–40]. Some PFAAs are toxic [23], bioaccumulative [41], and consistently occur in wastewater dominated systems [11]. Thus, detailed data about the occurrence, composition, and concentrations of PFAAs in water bodies entering preservation areas is needed to understand the potential risk of chronic exposure and devise strategies to reduce their impact on organisms inhabiting these areas.

### Longitudinal and Seasonal Distribution of PFAAs in the Water Column from Source to Sink

Of the 11 PFAAs analyzed in water samples, seven were detected in concentrations ≥1 ng/L in both seasons (S1 Table). The most frequently detected PFAAs were PFOS > PFOA > PFHxA > PFHpA > PFDA > PFNA. PFAAs with 11 to 14 C backbones were detected in concentrations up to 1 ng/L and the smallest PFAAs in terms of C backbone (perfluoro butanoate -4 C-) were detected at concentrations up to 2 ng/L.

**Fig 2:**
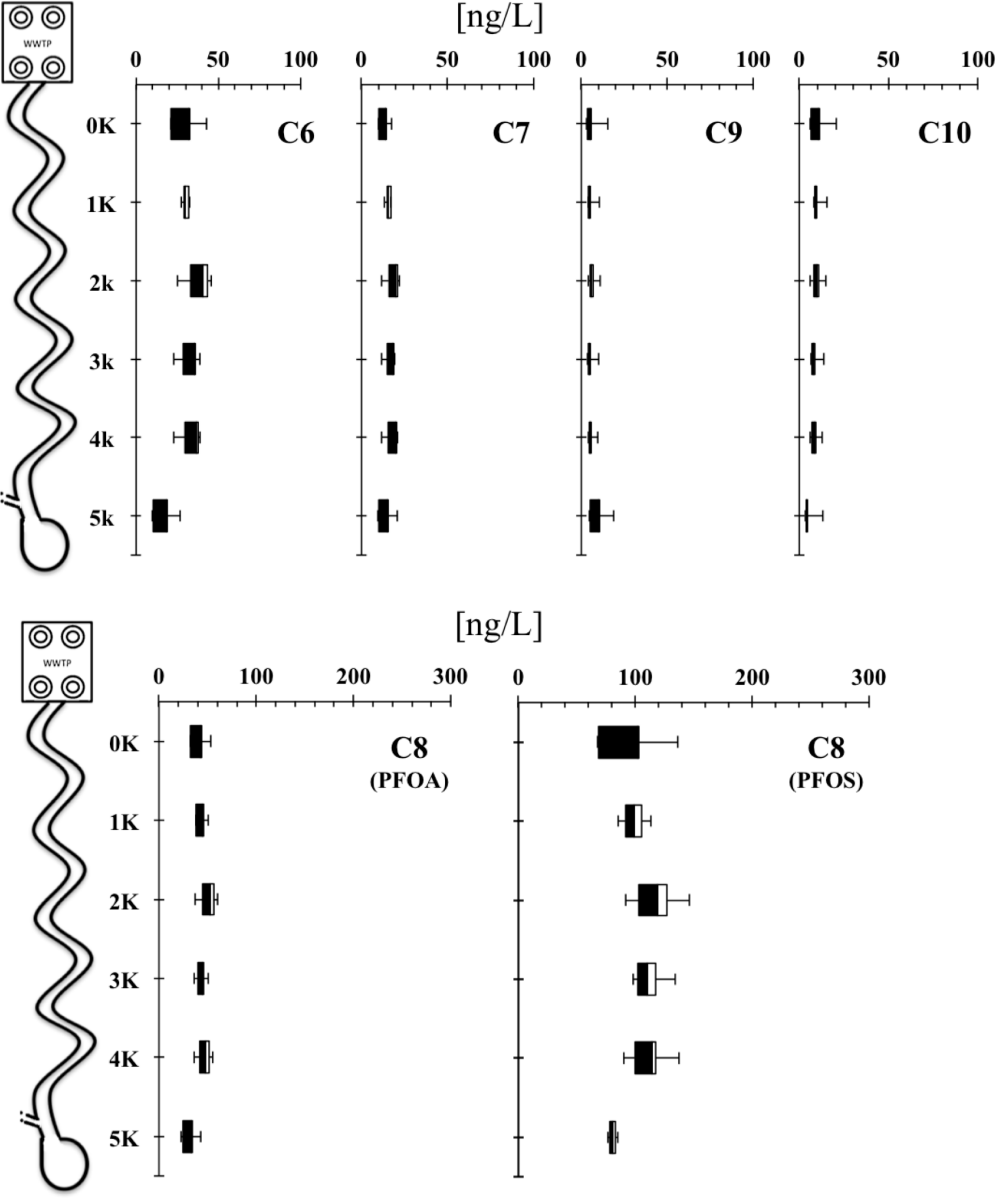
Longitudinal concentrations of PFAAs during the wet season. Box plot indicate minimum and maximum (bars), 225 quartile (white box), median (horizontal line), and 75 quartile (black box). No statistical differences were found for collections during the wet season.

During the wet season, the concentrations of detected PFAAs (mean ∑PFAAs = 196.6 ng/l) were similar (p = 0.343) at all sampling points with a slight reduction at the sinkhole site (Fig 2). PFOS and PFOA accounted for ≈50% and ≈20% of all measured PFAAs during the wet season, respectively (Table 2). The most frequently detected PFAAs showed a consistent trend at all sampled points, with a small reduction at the last (sinkhole) sampling point. The trend for two most concentrated PFAAs was very consistent longitudinally. For example, during the wet season, the mean PFOA concentration decreased from 42 ng/L to 29 ng/L at the sinkhole point (about 30% decrease). PFOS concentrations decreased from 103 ng/L to 79 ng/L, roughly a 23% decrease at the sinkhole point (S1 Table). We hypothesize that this 23 to 30% reduction at the sinkhole point is likely due to the dilution with PFAAs free water from the Camp’s canal (part of Newman’s Lake effluents), which runs through the preservation area (Fig 1). In the past, all of Newnan’s Lake flowed into Payne's Prairie but due to channelization about half of the flow now comes from this lake [30]. Currently, approximately 88% of the land use of the Newnan’s Lake basin is forest or wetlands with just a 5% of medium to low residential use [42]. Thus, we hypothesize that the contribution of water from Camp’s canal diluted the PFAAs concentration roughly by a factor of 25%; this corresponds well with our observed 23 to 30% reduction in PFAAs at sinkhole point.

**Table 2:** Total concentrations (ng/L) and percent of total PFAAs determined in the water column during the wet season at various longitudinal points in Sweetwater branch stream.

		% of Total PFAAs										
Site	∑PFAAs	C4	C6	C7	C8 (PFOA)	C8 (PFOS)	C9	C10	C11	C12	C13	C14
0K	200.7	0.68	15.06	6.62	20.29	49.50	2.32	5.25	0.18	0.07	0.01	0.01
1K	198.2	0.88	14.85	7.37	21.62	48.50	2.20	4.39	0.12	0.04	0.02	0.01
2K	217.8	1.58	15.01	7.29	20.79	49.10	2.24	3.81	0.11	0.04	0.03	0.01
3K	204.6	0.42	14.41	7.40	20.19	51.57	2.11	3.75	0.12	0.02	0.01	0.00
4K	206.5	0.77	14.42	7.59	20.37	50.74	2.26	3.57	0.18	0.06	0.02	0.01
5K (Sink)	151.5	1.68	10.15	8.67	19.30	51.96	5.40	2.40	0.09	0.10	0.16	0.08

**Fig 3:**
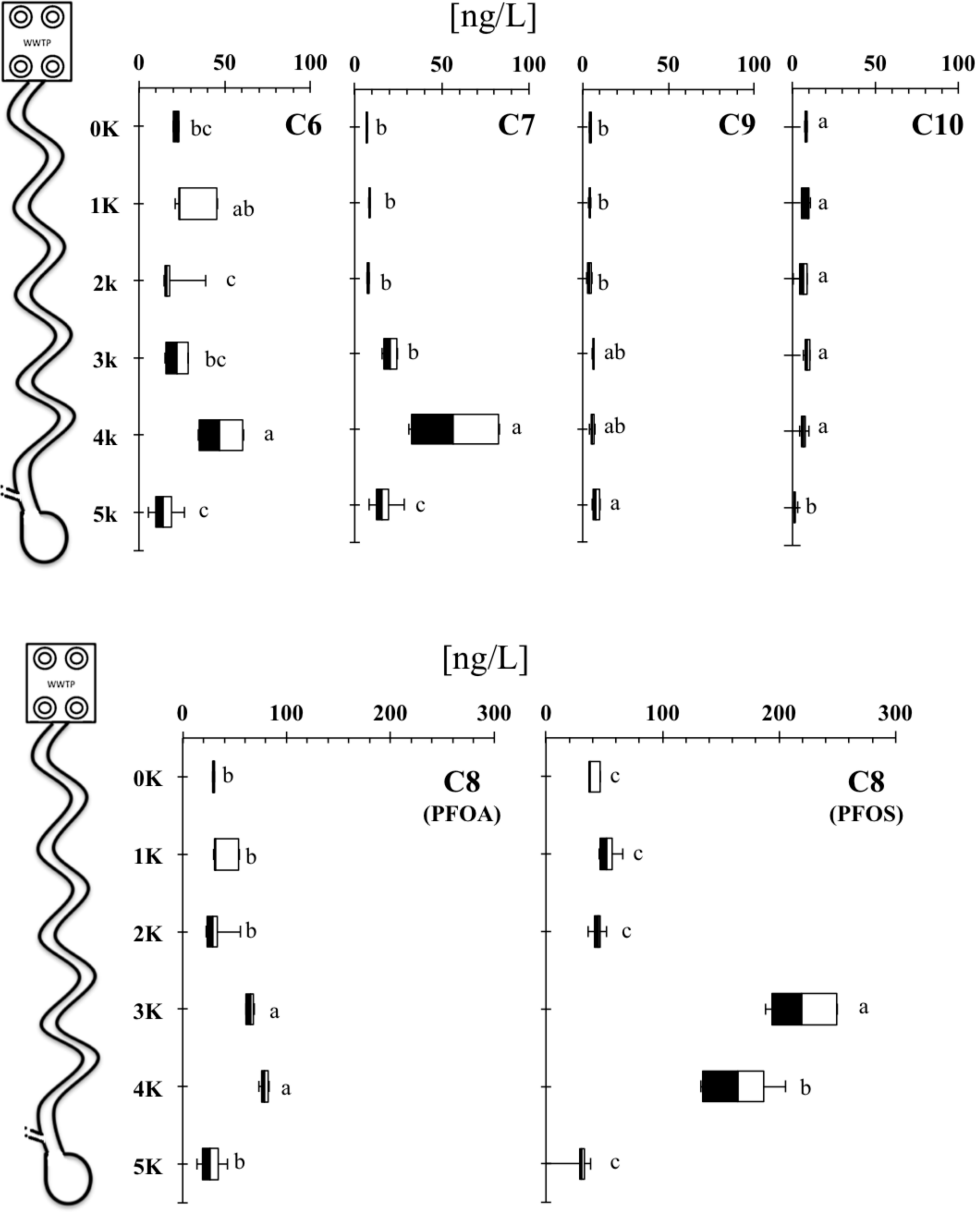
Longitudinal concentrations of PFAAs during the dry season. Box plot indicate minimum and maximum (bars), 25 quartile (white box), median (horizontal line), and 75 quartile (black box). Significantly different concentrations (ANOVA, *p*<0.05) among sites are shown with different letters.

In the dry season, the concentrations of detected PFAAs (mean ∑PFAAs = 200.7 ng/l) were statistically different at all sampling points with a slight reduction at the sinkhole site (Fig 3). PFOS and PFOA were ≈40% and ≈24% of all measured PFAAs, respectively (Table 3, Fig 3). Concentrations of some PFAAs (e.g., PFOS, PFOA, C7) at the 3 km and 4 km sampling points were 2- to 4-fold higher than the previous upstream sampling points at 0 and 1 km (Fig 3, S1 Table), but these did not influence the concentrations at the sinkhole. The higher concentrations at two sampling points (3 and 4 km) may be due to the diversion of all Sweetwater branch stream water as there are ongoing water flow restoration efforts around these points. The proposed “Sweetwater Branch Sheet Flow Restoration Project” is designed primarily to reduce the excess loads of total nitrogen, total phosphorus, and total suspended solids entering the Payne’s Prairie via Sweetwater branch, as part of compliance with total maximum daily loads [43]. Currently, several stages of this restoration project have been completed, including the construction of a wetland cell (an artificial wetland) in order to restore the historical sheet flow in Payne’s Prairie basin. Oddly, these “abnormal” concentrations (consistent between sampling period and replicates) showed an increase of 50% PFAAs with 8 or 9 C atoms. We observed that the amount of water in the stream at those two points was very low during the two dry season samplings (almost completely dry). Thus, due to the diversion of the water in this stream, the remnant stagnant water could evaporate resulting in concentrating some PFAAs such as 8 and 9 C PFAAs that do not volatilize [11]. Moreover, during the dry season, channel evaporative effects in rivers located in subtropical climates such as the central Florida can be higher and may influence the river water budgets. Further, the evaporative effects may be amplified when the channel flow decrease due to the changes in the surface area and channel volume ratio in the river [44]. Similar to wet season, the PFAAs concentrations from sampling point at 5 km were lower compared to the 3 km and 4 km samples (Fig 3) probably due to the dilution with contributing water from Camp’s canal, as discussed above (p<0.05).

**Table 3:** Total concentrations (ng/L) and percent of total PFAAs determined in the water column during the dry season at various longitudinal points in Sweetwater branch stream.

		% of Total PFAAs										
Site	∑PFAAs	C4	C6	C7	C8 (PFOA)	C8 (PFOS)	C9	C10	C11	C12	C13	C14
0K	128.2	1.52	18.88	9.41	25.43	35.04	3.34	6.15	0.16	0.05	0.01	0.01
1K	162.4	0.85	20.00	16.40	25.38	30.52	2.45	4.23	0.11	0.04	0.01	0.01
2K^DP^	106.8	0.66	15.35	7.07	26.72	40.33	3.73	5.89	0.19	0.05	0.01	0.02
3K*	348.8	0.29	6.35	5.79	18.44	64.49	1.78	2.64	0.15	0.05	0.01	0.01
4K*	357.1	0.33	13.47	16.34	22.30	43.80	1.60	1.97	0.13	0.04	0.01	0.01
5K (Sink)	100.9	0.75	14.93	16.28	27.30	31.41	7.87	0.94	0.22	0.08	0.18	0.04

^DP^: Diversion Point.

* Stagnant water.

Overall, PFAAs with an even number of C were dominant in water samples at all sampling points. Simcik and Dorweiler [45] reported that the ratio of perfluoro heptanoic acid (PFHpA) to PFOA can be used to infer the sources of PFAAs such as atmospheric or urban in aquatic systems. For example, if the ratio of PFHpA: PFOA is >1, then atmospheric deposition (rainfall) is the main source of PFAAs and if the ratio is <1, the majority of PFAAs originated either from runoff or wastewater sources. In this study, the PFHpA:PFOA at all sampling points was <1, suggesting that the sources of PFAAs are non-atmospheric [45].

Overall, PFOS was more dominant in the stream and was detected in relatively high concentrations as compared to the average global concentrations found in wastewater dominant streams [11]. However, globally PFOA (not PFOS) tends to be dominant in the water [46]. This contradicts the results from others researchers such as Pan et al. [16]; Guo et al. [15]; and Schultz et al. [8] who found that PFOS concentrations tend to decrease, while PFOA tends to increase after passing through WWTPs. Kim and Kannan [13] investigated the amount of PFAAs in snow, rain, surface runoff and lakes, and reported that PFOA was dominant in all environmental matrices. Why is PFOS more prevalent in our stream? One potential explanation could be the contribution of stormwater runoff from urban and commercial areas that drain Sweetwater branch (Fig 1). Xiao et al. [47] found that in runoff from urban areas, PFOS was dominant followed by PFOA. Thus, runoff contribution can explain why the concentrations of PFAAs in general are greater during the wet season and why PFOS is even more prevalent than PFOA during wet season. Also, unlike PFOS which is better correlated with WWTPs or urban activities, PFOA concentrations are heavily influenced by point source type emissions from industrial activities [48].

### Seasonal Variability in PFAAs Concentrations

No statistical significant differences were detected between seasons (*p* = 0.958). The main seasonal differences among all PFAAs in our study were related to the most predominant PFAAs determined in the water column. For example, mean and median PFOS concentrations were 99 ng/L and 93 ng/L during the wet season and 96 ng/L and 49 ng/L during the dry season, respectively. PFOA was the second most dominant PFAAs, with mean and median concentrations of 41 ng/L and 39 ng/L during the wet season and 47 ng/L and 34 ng/L during the dry season, respectively. If the abnormally high concentration points (at 3 km and 4 km) that we surmise are due to the water diversion in the canal are taken out of the calculation, important seasonal differences can be observed between wet and dry seasons (p < 0.001). In this case, all measured PFAAs are statistically different from each other in the wet and dry seasons, except for PFNA. The average dry season PFOS and PFOA without considering the abnormal monitoring points is 42 ng/L and 31 ng/L, respectively (as compared to 99 ng/L and 40 ng/L for the wet season). Thus, the difference between wet and dry season are marked mainly for PFOS with concentrations in the wet season that represent more than 50% of the dry season. Seasonal trends of PFAAs concentration in surface waters are not common findings. For example, Sakurai et al. [49] found no seasonal trends (average PFOS and PFOA 12 and 3.7 ng/L) in a 2-year study in Tokyo, Japan. In another study from Japan, Tsuda et al. [50] found seasonal differences for PFOS and PFOA, but just in 2 out of 14 sampling sites. Average annual precipitation for the Gainesville city area is 121 cm (NOAA, 1981 to 2010 [29]) and more than half of this amount falls from June to September. The higher concentrations particularly of PFOS during wet season are likely due to the stormwater runoff from urban parts of Gainesville city.

Comparing the mean and median concentrations of PFOS and PFOA in our system with others (e.g., [11, 46]), it is clear that our stream receives higher amounts of PFAAs. For example, in the WWTP effluents, the reported PFOS and PFOA median concentrations are 11 ng /L and 24 ng/L, respectively [46]. Thus, the median concentrations of PFOS and PFOA in our study site were 8- and 1.6- times greater during the wet season and 4.5- and 1.4- times greater during the dry season, respectively. Contribution of PFAAs during the wet season may be related with street stormwater runoff. Zushi and Masunaga [51] analyzed the first flush loads of PFAAs and observed higher loads of long length (10 to 12 C) but no change in concentrations of short to medium length (4 to 9 C) PFAAs. In our study, the main change during the wet season was driven by the higher loads of the medium length PFOS (Table 2, Fig 2). Others authors [52, 53] also find no change in PFOS concentrations due to runoff. Nakayama et al. [54] found that PFOS was the PFAAs with the second highest concentration (245 ng/L) and the highest median concentration (3.0 ng/L) in water from the upper Mississippi basin suggesting a non-point source influence. However, they showed that increased flow did not increase PFOS and suggested that firefighting foams were a potential PFOS source. On the contrary, Xiao et al. [47] found that runoff from industrial and commercial areas, especially the food packaging from restaurants, had higher levels of PFOS. Moreover, these same authors [47] found that solid particles in stormwater runoff contributed substantially to higher concentrations of PFOS. In general, our water samples were not particle free, but with an amount of particles that allowed us to extract the sample without any filtration. Since we did not use a filtration step or discard the particle fraction before the SPE, it is plausible that we caught both free and particle-bond PFOS. Another potential source may be related with landfill leachate, as it is known that PFAAs can leach from landfills [6–8]. The Sweetwater branch is a tributary of the Tumblin Creek, the later stream passes through an old landfill site [42]. Therefore, the presence of this landfill may also explain the relatively high concentrations of PFAAs found in our study.

### PFAAs Concentrations in Sediments

The physical and chemical characteristics of sediments such as texture, pH, iron, and organic matter can provide insights on PFAAs sorption behavior and can be used to determine the amount of PFAAs in sediments [19, 55]. It is known that PFAAs (i.e. PFOS and PFOA) sorption to solids is very weak with distribution coefficients normalized by the organic C content (K_oc_) between 2 to 3 [10]. In this study, sediments had 7.96 to 8.34 pH (Table 4) and less than 1% organic matter (OM). The texture of sediment samples ranged from loamy sandy to sandy, with 70–98% sand, (Table 4) with no particular pattern related to the three different depths. Also, the separation of sediments into three depths was not possible at the point 0 (downstream of WWTP) due to the high sand content. The extract iron concentration determined in sediments (Table 4) was in the range of 108–344 mg/kg, while the concentrations of nutrients and chloride (S1 Table) were variable.

**Table 4:** Organic Mater, texture, pH, and iron at three depths in sediments from Sweetwater branch canal.

Site	Depth (cm)	pH	OM%	% Sand	% Silt	% Clay	Fe (mg/kg)
0K	0–5	8.3	0.2	96.8	0.5	2.7	154.0
3K	0–5	8.2	0.3	70.3	23.0	6.7	297.6
3K	5–10	8.0	0.7	75.3	22.0	2.7	332.9
3K	10–15	8.1	0.1	58.3	38.0	3.7	344.8
5K	0–5	8.3	0.2	98.3	0.0	1.7	108.3
5K	5–10	8.1	0.2	98.3	0.0	1.7	151.9
5K	10–15	8.0	0.2	98.3	0.0	1.7	171.9

Sweetwater branch is a highly modified stream with some channelized sections that results in different composition (and texture) of sediments, which may explain the variability in nutrient content among sampling sites and depths. No PFAAs were determined in concentrations higher than the limit of quantification (LOQ: 0.1–2.5 ng/g) in sediments at any depth. Determination of partition coefficient and sorption are important to verify the environmental fate of PFAAs [55, 56]. A factor affecting the partitioning is the organic C content, as increasing PFAAs sorption has been observed with increasing organic C content [19]. For example, Theobald et al. [56] found the highest PFAAs concentrations at sites with high silt and C; opposed to the lowest concentrations in sandy sediments, suggesting a strong influence of sediment C on PFAAs concentrations. It is difficult to compare concentrations of PFAAs in sediments from different locations without knowing the C content. The texture characteristic and OM content (Table 4) appear as the most plausible causes for the low concentration of PFAAs determined in our sediments. Overall, the data suggest that the low content of OM, high pH, and high content of sand in sediments likely affected the ability of sediments to sorb and accumulate PFAAs.

PFAAs have been determined mainly in biota and water samples. Few studies have been conducted on PFAAs in sediments [56]. The role of sediments in the fate of PFAAs may be more important than expected. In general, enrichment of PFAAs from water to sediment is moderate. As a result, suspended matter and sediments may act as sinks for PFAAs such as PFOS [22]. As indicated by other authors [20, 57], the role of sediment partitioning and cycling must be better understood to consider the overall fate of PFAAs in the aquatic systems.

### Final Considerations for Ecological and Human Health

PFAAs have been detected in the wildlife worldwide [5, 12, 58] and are thought to be the most prevalent contaminant in human blood [59]. Some researchers such as Theobald et al. [56] do not recommend the use of sediments to determine the fate of PFAAs as the first-choice matrix because sediment parameters like C strongly influence the enrichment process in addition to the high spatial variability not related to pollution. In contrast, the water phase could be a better matrix for investigating the fate and distribution of PFAAs concentrations because it allows the identification of sources. Sediments are in general a good matrix to study the occurrence of PFAAs when sufficient amounts of C and small particle fractions (clay, silt) are present. The absence of PFAAs in our sediment samples is attributed to low OM (and C) and high sand contents. This implies that sediment samples with low C and high sand content may not be an ideal matrix to investigate the fate of PFAAs, as also suggested by others [56]. We were able to determine PFAAs in the water column at all sampling periods at relatively high concentrations in contrast with the low concentrations of PFAAs (under the limit of quantification) in sediments because of the low C and high sand content of the sediments.

The toxic effects of some PFAAs in the aquatic biota have been recognized (reviewed by Lau [40]) but most of the literature considers that the typical concentrations found in the aquatic environment are too low to be considered a risk for aquatic biota. For example, Lin et al. [60] discussed that high levels of PFAAs (e.g., PFOS 293 ng/L; perfluorohexanoic acid 406 ng/L), which are at least twice the levels found here, pose a risk for aquatic communities downstream of WWTPs. But Newsted and collaborators [61] suggested that 43 ng/L of PFOS is a safe water concentration that is protective of avian wildlife, a value that is considered to be overly conservative [62]. Our research determined PFOS concentrations over this threshold (as high as five times) in the two seasons from all sampling points except for the sinkhole site (due to dilution). Moreover, the traditional approach to determine toxicity does not take into consideration the sub-chronic effects or more sensitive endpoints such as gene expression changes or the synergistic effects of contaminant mixtures typically found in wastewater dominated systems. The new approaches to evaluate toxicity are lowering the concentrations of contaminant that are considered dangerous. For instance, our previous work in the Sweetwater branch reported in Rodriguez-Jorquera et al. [28] found that the effects of PFAAs in wastewater dominated systems can alter important gene transcription patterns and cholesterol physiology in fish.

Further, as protected areas are set aside to maintain biodiversity, special consideration should be taken to protect these areas. Surprisingly, research on aquatic pollution impacts on protected areas are rarely, if not completely, absent in the scientific literature. The occasional research papers that discuss pollution pressure in protected areas highlight the impact of the types of pollution that apparently are less obvious and harmful, such as light, air, and noise pollution (i.e. [24, 63]). Some exceptions like Mora and Sale [64] mention pollution as threat and suggest the increased use of WWTPs as a solution, nevertheless, our research demonstrated that typical technologies used in WWTPs are not sufficient to remove some micro-pollutants such as PFAAs.

Considering the current biodiversity crisis particularly in freshwater ecosystems [65], the known impact of pollutants on wildlife, and the recognized ability of preserved areas to conserve biodiversity, understanding of the presence (as well as the toxicity) of pollutants such as PFAAs inside preservation areas is of the utmost importance. For example, other contaminants were also present in the historical water sampling campaigns in Sweetwater branch (see [28]). It is striking to find consistent presence of pharmaceutical compounds such as carbamazepine, diazepam, and fluoxetine, which target the nervous system of animals. Our previous work, reported in Yang et al. [66] investigated pharmaceuticals in sediments in an urban river in Florida and found that carbamazepine was the most prevalent compound present in 100% of sediments samples. The high persistence of some pharmaceuticals such as carbamazepine [67] may explain why these types of micro-pollutants are commonly found in wastewater dominated systems. Overall, to protect wildlife protected areas from urban micro-pollution, we need a comprehensive understanding of various pollutants that not necessarily occur at high levels. It is only then we can develop and implement practices to reduce the continuous exposure of organisms to micro-pollution inside protected areas.

## Conclusions

PFAAs were detected in the water column along all stream sampling sites with a minor reduction in concentration in the sinkhole point, likely due to the dilution. The most detected PFAAs were PFOS>PFOA>PFHxA>PFHpA>PFDA>PFNA. Minor seasonal differences in PFAAs concentrations were found. Among all PFAAs measured, PFOS was the most prevalent accounting for 40–50%. The PFHpA to PFOA ratio at all sites was <1, suggesting that wastewater and urban stormwater runoff were the major sources of PFAAs. Two points downstream of the restoration project showed higher concentrations of PFOS (156 and 225 ng/L) and PFOA (64 and 80 ng/L), but further research is needed to determine the cause of this phenomenon. Despite the constant occurrence of PFAAs in water samples, the concentration of PFAAs determined in the sediments was under the limit of quantification. We demonstrated that relatively toxic micro-pollutants such as PFAAs were consistently present throughout the year inside two protected areas. Overall, this system receives relatively high amounts of PFAAs compared to global concentrations and cities with similar size. As concentrations of some PFAAs found in this study such as PFOS (224 ng/L) are near levels considered dangerous for aquatic biota (i.e. PFOS: 294 ng/L, [60]) or over the concentrations considered safe for wildlife birds (PFOS > 43 ng/L, [61]), continuation of this type of research inside preservation areas around the world is recommended to track the footprints of urban micro-pollution and achieve protection of target and endangered species.

## Supporting Information

S1 Appendix Contains the abstract in Spanish language. (DOCX)

S1 Table Contains s with additional information including the Multiple Reaction Monitoring (MRM) and its Transitions numbers, limits of determination (LOD) and limit of quantification (LOQ) for PFAAs in water, all determined PFAAs in water and parameters for sediments such as metals, nutrients and chloride. (DOCX)
