# Supplementary material for: Footprints of Urban Micro-Pollution in Protected Areas: Investigating the Longitudinal Distribution of Perfluoroalkyl Acids in Wildlife Preserves
Source: PLoS One. 2016 Feb 24;11(2):e0148654. doi: 10.1371/journal.pone.0148654 (PMC4766195; doi:10.1371/journal.pone.0148654)
Supplement: S1 Appendix — (DOCX) [file pone.0148654.s001.docx]

**S1 Appendix**

**Alternative Language Spanish (Español)**

Los enfoques actuales para proteger la biodiversidad mediante el establecimiento de áreas protegidas por lo general no consideran la contaminación del agua como una amenaza. Nuestro objetivo fue determinar la distribución longitudinal y estacional de acidos perfluoro-alquilados (PFAAs, por sus siglas en inglés) en el agua y los sedimentos de un río que recibe aguas servidas (residuales) y que pasa por dos áreas protegidas. Las muestras de agua fueron recolectadas a lo largo del río (seis sitios, a 1.000 metros de distancia el uno del otro) durante las estaciones seca y lluviosa. Los sedimentos se obtuvieron en tres sitios desde el centro del lecho del río a tres profundidades. Se analizaron las muestras de agua y de sedimentos para PFAAs utilizando cromatografía líquida de alta resolución y espectrometría de masas en tándem. Once PFAAs de 5 a 14 átomos de carbono de longitud fueron detectados en la columna de agua en todos los puntos de muestreo, con una ligera reducción en su concentración en el último punto de muestreo, lo que sugiere un efecto de dilución. Los PFAAs más detectadas en el agua fueron PFOS, seguido por el ácido perfluoro-octanoico (PFOA), y ácido perfluoro-hexanoico (PFHxA). Diferencias estacionales en las concentraciones PFAAs fueron determinadas lo que sugiere una contribución de la escorrentía de aguas pluviales durante la estación húmeda. Todas las PFAAs analizados en sedimentos estaban bajo el límite de cuantificación, probablemente debido a la alta proporción de arena y baja proporción de materia orgánica en los sedimentos del lugar. Sin embargo, se detectaron altas concentraciones de PFAAs en la columna de agua dentro de las áreas protegidas, incluyendo PFOS en concentraciones consideradas como no seguras para las aves. Las muestras de agua fueron mas útiles que los sedimentos para determinar la micro-contaminación por PFAAs en cuerpos de agua con sedimentos arenosos. Se recomienda la inclusión de un plan de investigación y gestión, vigilancia y mitigación de la micro-contaminación dentro de estas áreas protegidas de manera de evitar su impacto en la biota.
