# Supplementary material for: Footprints of Urban Micro-Pollution in Protected Areas: Investigating the Longitudinal Distribution of Perfluoroalkyl Acids in Wildlife Preserves
Source: PLoS One. 2016 Feb 24;11(2):e0148654. doi: 10.1371/journal.pone.0148654 (PMC4766195; doi:10.1371/journal.pone.0148654)
Supplement: S1 Table — (DOCX) [file pone.0148654.s002.docx]

S1 Tables

Supporting Information

**S1 Table. Perfluoroalkyl acids analyzed by Multiple Reaction Monitoring (MRM) and its Transitions numbers, limits of determination (LOD) and limit of quantification (LOQ) in water samples collected from Sweetwater branch.**

| **ID** | **MRM transitions ^-^(m/z)** | **LOD (ng/L)** | **LOQ (ng/L)** |
| --- | --- | --- | --- |
|  |  |  |  |
| PFBA | 213→169 | 0.01 | 0.032 |
| PFHxA | 313→269 | 0.009 | 0.02 |
| PFHpA | 363→319 | 0.01 | 0.032 |
|  | 363→169 |  |  |
| PFOA | 413→219 | 0.009 | 0.028 |
|  | 413→169 |  |  |
| PFNA | 463→419 | 0.009 | 0.029 |
|  | 463→219 |  |  |
| LPFOSK | 499→80 |  |  |
|  | 499→130 | 0.01 | 0.026 |
|  | 499→169 |  |  |
|  | 499→330 |  |  |
|  | 499→419 |  |  |
| PFDA | 513→469 | 0.009 | 0.037 |
|  | 513→269 |  |  |
| PFUdA | 563→269 | 0.01 | 0.035 |
|  | 563→219 |  |  |
| PFDoA | 613→569 | 0.009 | 0.024 |
|  | 613→319 |  |  |
| PFTrDA | 663→619 | 0.01 | 0.033 |
|  | 663→319 |  |  |
| PFTeDA | 713→669 | 0.01 | 0.026 |
|  | 713→169 |  |  |

**S2 Table. Average +/- SD of PFAAs concentrations (ng/L) determined at various longitudinal points downstream of a WWTP in Sweetwater branch during the 2012 wet season.**

| **PFAAs**  **Type** | **Distance from WWTP (Km)** | | | | | | | | | | | | | | | | |
| --- | --- | --- | --- | --- | --- | --- | --- | --- | --- | --- | --- | --- | --- | --- | --- | --- | --- |
|  | Point 0K | |  | 1K | |  | 2K | |  | 3K | |  | 4K | |  | 5K (Sink) | |
| C4 | 1.37 | ±0.58 |  | 1.75 | ±0.48 |  | 3.44 | ±3.65 |  | 0.85 | ±0.45 |  | 1.58 | ±0.77 |  | 2.55 | ±2.82 |
| C6 | 30.23 | ±11.02 |  | 29.43 | ±2.72 |  | 32.69 | ±10.95 |  | 29.48 | ±8.27 |  | 29.77 | ±7.88 |  | 15.38 | ±9.53 |
| C7 | 13.28 | ±3.97 |  | 14.61 | ±2.01 |  | 15.87 | ±5.62 |  | 15.14 | ±4.17 |  | 15.68 | ±4.68 |  | 13.14 | ±6.29 |
| PFOA | 40.71 | ±10.72 |  | 42.86 | ±7.17 |  | 45.27 | ±11.97 |  | 41.31 | ±7.30 |  | 42.07 | ±7.01 |  | 29.24 | ±10.80 |
| PFOS | 99.35 | ±36.40 |  | 96.13 | ±14.03 |  | 106.93 | ±27.55 |  | 105.54 | ±18.19 |  | 104.78 | ±21.19 |  | 78.74 | ±4.20 |
| C9 | 4.66 | ±1.66 |  | 4.36 | ±0.46 |  | 4.88 | ±1.48 |  | 4.31 | ±0.96 |  | 4.66 | ±0.89 |  | 8.19 | ±6.02 |
| C10 | 10.54 | ±4.90 |  | 8.71 | ±1.38 |  | 8.3 | ±3.02 |  | 7.67 | ±1.51 |  | 7.38 | ±1.70 |  | 3.64 | ±0.78 |
| C11 | 0.36 | ±0.15 |  | 0.24 | ±0.08 |  | 0.25 | ±0.01 |  | 0.25 | ±0.11 |  | 0.37 | ±0.05 |  | 0.13 | ±0.08 |
| C12 | 0.14 | ±0.10 |  | 0.07 | ±0.04 |  | 0.09 | ±0.06 |  | 0.05 | ±0.01 |  | 0.13 | ±0.07 |  | 0.15 | ±0.14 |
| C13 | 0.03 | ±0.02 |  | 0.04 | ±0.01 |  | 0.06 | ±0.05 |  | 0.03 | ±0.02 |  | 0.04 | ±0.02 |  | 0.25 | ±0.36 |
| C14 | 0.02 | ±0.01 |  | 0.02 | ±0.01 |  | 0.02 | ±0.01 |  | 0.01 | ±0.01 |  | 0.03 | ±0.03 |  | 0.12 | ±0.15 |

The letter *“C”* represent the size (number of carbons) in Perfluoroalkyls Acids. *PFOS*: Perfluorooctane Sulfonate.

**S3 Table. Average +/- SD of PFAAs concentrations (ng/L) determined at various longitudinal points downstream of a WWTP in Sweetwater branch during the 2013 dry season.**

| **PFAAs**  **Type** | **Distance from WWTP (Km)** | | | | | | | | | | | |
| --- | --- | --- | --- | --- | --- | --- | --- | --- | --- | --- | --- | --- |
|  | Point 0K | | 1K | | 2K (Diversion Point) | | 3K | | 4K | | 5K (Sink) | |
| C4 | 1.95 | ±1.05 | 1.38 | ±0.75 | 0.71 | ±0.14 | 1.02 | ±0.42 | 1.18 | ±0.50 | 0.76 | ±0.31 |
| C6 | 24.2 | ±3.93 | 32.48 | ±27.19 | 16.4 | ±1.78 | 22.14 | ±7.56 | 48.1 | ±17.82 | 15.06 | ±6.09 |
| C7 | 12.06 | ±15.76 | 26.62 | ±38.72 | 7.55 | ±0.92 | 20.21 | ±4.80 | 58.35 | ±31.97 | 16.43 | ±5.13 |
| PFOA | 32.6 | ±1.30 | 41.2 | ±29.17 | 28.54 | ±6.10 | 64.33 | ±5.13 | 79.62 | ±8.39 | 27.55 | ±10.03 |
| PFOS | 44.92 | ±12.78 | 49.56 | ±24.71 | 43.08 | ±5.39 | 224.91 | ±37.34 | 156.42 | ±40.69 | 31.69 | ±4.51 |
| C9 | 4.28 | ±1.64 | 3.97 | ±1.38 | 3.98 | ±1.45 | 6.21 | ±0.79 | 5.71 | ±1.21 | 7.94 | ±2.71 |
| C10 | 7.89 | ±2.27 | 6.87 | ±4.32 | 6.29 | ±2.77 | 9.21 | ±2.71 | 7.05 | ±2.02 | 0.95 | ±0.24 |
| C11 | 0.21 | ±0.09 | 0.18 | ±0.06 | 0.2 | ±0.13 | 0.51 | ±0.23 | 0.47 | ±0.10 | 0.22 | ±0.16 |
| C12 | 0.06 | ±0.01 | 0.06 | ±0.04 | 0.05 | ±0.01 | 0.16 | ±0.12 | 0.13 | ±0.07 | 0.08 | ±0.03 |
| C13 | 0.01 | ±0.01 | 0.02 | ±0.02 | 0.01 | ±0.01 | 0.05 | ±0.02 | 0.05 | ±0.02 | 0.18 | ±0.21 |
| C14 | 0.01 | ±0.01 | 0.02 | ±0.01 | 0.02 | ±0.01 | 0.02 | ±0.01 | 0.04 | ±0.02 | 0.04 | ±0.03 |

The letter *“C”* represent the size (number of carbons) in Perfluoroalkyls Acids. *PFOS*: Perfluorooctane Sulfonate.

**S4 Table. Concentrations (mg/Kg) of magnesium (Mg), aluminum (Al), and boron (B) at three depths in sediments collected from three longitudinal points downstream of a WWTP in Sweetwater branch.**

| **Site** | **Depth (cm)** | **Mg** | **Al** | **B** |
| --- | --- | --- | --- | --- |
| 0K | 0-5 | 65.3 | 133.2 | 0.2 |
| 0K | 0-5 | 138.7 | 186.4 | 0.0 |
| 3K | 0-5 | 959.8 | 576.4 | 0.3 |
| 3K | 5-10 | 842.2 | 327.4 | 0.1 |
| 3K | 10-15 | 873.9 | 497.8 | 0.1 |
| 5K | 0-5 | 87.6 | 31.7 | 0.3 |
| 5K | 5-10 | 113.6 | 24.7 | 0.0 |
| 5K | 10-15 | 69.3 | 13.3 | -0.1 |

**S5 Table. Concentrations (mg/Kg) of nutrients and chloride (Cl) at three depths in sediments collected from three longitudinal points downstream of a WWTP in Sweetwater branch.**

| **Site** | **Depth (cm)** | **Cl** | **P** | **K** | **Ca** | **NOx-N** | **NH_4_-N** | **TKN** |
| --- | --- | --- | --- | --- | --- | --- | --- | --- |
| 0K | 0-5 | 21.23 | 95.26 | 16.10 | 1203.67 | 1.68 | 3.60 | 43.70 |
| 0K | 0-5 | 29.79 | 108.01 | 24.89 | 1625.93 | 2.21 | 1.91 | 65.67 |
| 3K | 0-5 | 43.01 | 96.74 | 118.44 | 2869.94 | 3.20 | 3.98 | 138.05 |
| 3K | 5-10 | 28.15 | 177.81 | 106.64 | 4767.59 | 2.12 | 4.88 | 408.24 |
| 3K | 10-15 | 18.27 | 196.29 | 90.75 | 2720.11 | 29.35 | 5.49 | 178.23 |
| 5K | 0-5 | 23.93 | 85.23 | 21.67 | 1654.76 | 2.95 | 3.32 | 66.57 |
| 5K | 5-10 | 32.49 | 104.83 | 28.72 | 2593.50 | 2.74 | 5.17 | 99.44 |
| 5K | 10-15 | 28.96 | 97.34 | 29.90 | 3087.83 | 7.63 | 3.59 | 109.02 |

P=phosphorus, K: potassium, Ca: calcium, NOx-N: nitrate-nitrogen, NH_4_-N: ammonium-nitrogen, TKN: total kjeldahl nitrogen.
